# Distal cholangiocarcinoma with rare progression to distal femoral metastasis: a case report and literature review

**DOI:** 10.3389/fmed.2026.1776401

**Published:** 2026-04-20

**Authors:** Ning Du, Yanchao Gao, Huidong Sun, Xueli Zhang

**Affiliations:** Department of Hepatobiliary Surgery, Liaocheng People’s Hospital, Liaocheng, Shandong, China

**Keywords:** bone metastasis, cholangiocarcinoma, femoral metastasis, prognosis, treatment

## Abstract

Cholangiocarcinoma (CCA) is a malignant neoplasm arising from the biliary epithelium and is associated with an exceptionally poor prognosis. Metastasis to long bones is particularly uncommon. A 66-year-old male patient developed femoral metastasis 2 years following a pancreaticoduodenectomy for distal cholangiocarcinoma and subsequently underwent total knee arthroplasty. He experienced a satisfactory postoperative recovery, with a marked enhancement in mobility. This case suggests that radical surgical intervention may be a viable option for metastatic bone disease characterised by isolated lesions. As illustrated in this instance, this therapeutic approach can alleviate symptoms and enhance the quality of life for the remaining duration.

## Introduction

Cholangiocarcinoma (CCA) is a lethal biliary epithelial carcinoma that arises from the liver (intrahepatic), the confluence of the left and right hepatic ducts (hilar), or the extrahepatic bile ducts. This rare malignant tumor is associated with a dismal prognosis, with an annual incidence of 1–2 cases per 100,000 individuals in Western countries (1). Distal cholangiocarcinoma (DCC) constitutes approximately 30% of all cholangiocarcinomas. While radical surgical resection can provide a potential cure and promote long-term survival for select patients, the 5-year survival rate remains only between 18% and 54% even after R0 resection. This low overall survival rate is indicative of a high recurrence rate (2).

Most initial recurrences following cholangiocarcinoma surgery occur in the local region. The liver is the most common site for distant metastases, followed by the peritoneum. Numerous reports have documented metastases to the lungs, brain, adrenal glands, and axial skeleton (3, 4). Metastasis of cholangiocarcinoma to the extremities, particularly long bones, is exceedingly rare. To our knowledge, there are few pertinent reports in the literature (Table 1). Other researchers have described three cases of humeral metastasis due to cholangiocarcinoma (5–7), two of which were managed with surgical fixation, while the third received only palliative radiotherapy in conjunction with chemotherapy. Additionally, one report indicated that a patient was diagnosed with cholangiocarcinoma after presenting with distal fibular metastasis (8). Another case involved a patient with hilar cholangiocarcinoma who developed distal right femoral metastasis 6 years post-surgery and subsequently underwent distal femoral replacement (9). Most of these primary tumors were classified as intrahepatic cholangiocarcinoma (ICC). This case represents the first reported instance of femoral metastasis following R0 resection for distal cholangiocarcinoma. We consider this case to be of significant importance. Although the prognosis for this rare occurrence of long bone metastasis from cholangiocarcinoma is poor, major surgical interventions such as fixation and joint replacement may still alleviate pain, enhance mobility, and improve the quality of life for affected patients.

**Table 1:** Information of patients with cholangiocarcinoma metastasis to long bones.

First author, year	Carlisle and Roberts (5)	Lahrach et al. (6)	Federico et al. (7)	Karanjia et al. (8)	MacKenzie et al. (9)	Present case
Age, years	60	58	71	75	61	66
Sex	F	NA	M	F	F	F
Primary	ICC	NA	ICC	ICC	ICC	DCC
Metastatic site	Humerus	Humerus	Humerus	Fibula	Femur	Femur
Treatment	Intramedullary nail	Plate and screw osteosynthesis and bone cement filling	Chemotherapy radiotherapy	Radiotherapy	Distal femoral replacement	Total knee arthroplasty
Post-metastasis survival	>4 weeks	3 months	NA	>12 months	4 weeks	>6 months

F, female; NA, not available; M, male; ICC, intrahepatic cholangiocarcinoma; DCC, distal cholangiocarcinoma.

### Case presentation

We present an exceptionally rare case involving a 66-year-old male who was admitted to the hospital due to right knee joint pain persisting for over 2 weeks. Upon admission, his carcinoembryonic antigen (CEA) level was 17.37 ng/mL, carbohydrate antigen 19-9 (CA19-9) was 60.32 U/mL, and carbohydrate antigen 125 (CA125) was 49.7 U/mL. A physical examination revealed no abnormalities. Two and a half years prior, the patient underwent a pancreaticoduodenectomy for distal cholangiocarcinoma. Postoperative pathology confirmed the presence of moderately to poorly differentiated adenocarcinoma of the pancreatobiliary type, characterized as periductal invasive, with a tumor size of approximately 1.5 cm × 1 cm × 0.3 cm. The cancer had infiltrated the bile duct wall and invaded the adjacent pancreas and muscularis propria of the duodenum. Nerve invasion was noted; however, no typical intravascular tumor thrombus was identified. The resection margin was negative, and lymph nodes were also negative, resulting in an evaluation of R0 resection. The adenocarcinoma was staged as T3aN0M0, corresponding to stage IIA. Postoperatively, the patient received six cycles of comprehensive treatment consisting of gemcitabine, albumin-bound paclitaxel, and sintilimab, with no recurrence observed during postoperative follow-up.

**Figure 1:**
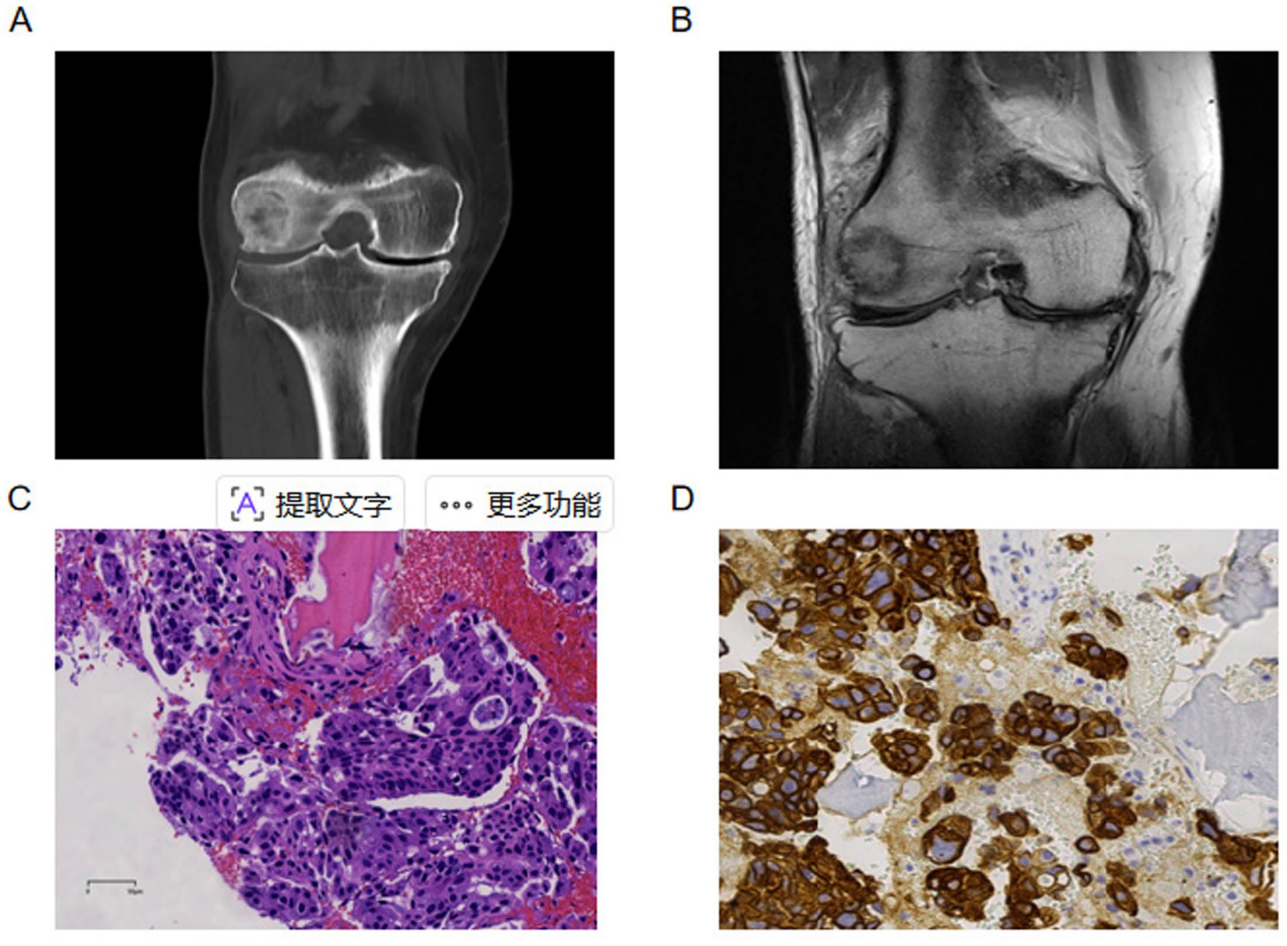
**(A)** Computed tomography (CT): bone destruction of the lateral condyle in the distal right femur. **(B)** Magnetic resonance imaging (MRI): abnormal signal in the lateral condyle of the distal right femur. **(C)** Tumor morphology of cholangiocarcinoma with bone metastasis (HE×200). **(D)** Immunohistochemistry: Cytokeratin 7 (CK7) positive (×200).

Computed tomography (CT) and magnetic resonance imaging (MRI) revealed bone destruction in the lateral condyle of the distal right femur (Figures 1A,B). Gastrointestinal endoscopy and enhanced thoracoabdominal CT did not show any abnormalities. The patient underwent a whole-body bone scan, which suggested a metastatic tumor in the distal right femur; no metastases were identified in other sites. The patient underwent a needle biopsy of the right femoral tumor, which confirmed the presence of metastatic carcinoma. Combined with immunohistochemical markers and a comparison with the original surgical specimen from the patient’s pancreaticoduodenectomy, the findings are consistent with metastatic cholangiocarcinoma. The immunohistochemical results from the biopsy pathology were as follows: CK7 (+), TTF-1 (−), Napsin A (−), P40 (partially +), NKX3.1 (−), CK20 (−), Villin (−), CK5/6 (+), Mucin1 (+), CK19 (+), P63 (partially +), PAS (+) (Figures 1C,D). The patient subsequently received two cycles of comprehensive antitumor treatment consisting of gemcitabine and albumin-bound paclitaxel, combined with sintilimab. Re-examination indicated a significant decrease in tumor markers: carcinoembryonic antigen (CEA) was 11.94 ng/mL, and carbohydrate antigen CA19-9 was 21.83 U/mL.

Due to the patient’s severe pain in the right knee joint and difficulty ambulating, a multidisciplinary team (MDT) comprising the Oncology, Hepatobiliary Surgery, Joint Surgery, Anesthesiology, Radiation Oncology, and Rehabilitation Medicine Departments convened to discuss the risks and benefits of surgical versus non-surgical treatment options. The patient opted for total knee arthroplasty. Given the patient’s favorable general condition, acceptable life expectancy, and high level of daily activity, the MDT considered this an appropriate treatment choice. The patient underwent total knee arthroplasty under general anesthesia. The surgery and subsequent rehabilitation proceeded without complications, and he was discharged successfully 1 week postoperatively. At the 8-week follow-up, tumor markers CEA and CA199 were within normal limits. The patient demonstrated normal mobility, and his pain was significantly alleviated compared to preoperative levels. Given the patient’s favorable postoperative recovery and the normalization of tumor markers, the patient declined comprehensive antineoplastic therapy. Notably, no tumor recurrence has been observed during the follow-up period of 6 months to date.

## Discussion

Cholangiocarcinoma (CCA) is a malignant tumor that arises from biliary epithelium and is associated with an exceedingly poor prognosis. Distant metastases frequently occur in the liver, lungs, peritoneum, and lymph nodes. The incidence of bone metastasis is relatively low, at only 1.51%, with most cases involving the axial skeleton, including the spine, ribs, and pelvis. Metastasis to long bones is particularly uncommon, with only a limited number of case reports documenting involvement of the humerus, fibula, femur, and other sites (10). The patient in this case was a 66-year-old male who developed distal right femoral metastasis 2 years following pancreaticoduodenectomy for distal cholangiocarcinoma (DCC) and subsequently underwent total knee arthroplasty. No tumor recurrence has been observed during follow-up to date, representing an exceptionally rare clinical scenario. To our knowledge, this is the first reported case of femoral metastasis following R0 resection for distal cholangiocarcinoma.

Two therapeutic strategies are available for managing bone metastasis following cholangiocarcinoma surgery: (1) Surgery-first strategy: This approach is preferred for patients with isolated long bone metastasis from cholangiocarcinoma who exhibit no distant organ involvement, maintain a favorable general condition, and have high demands for daily activities. This strategy can rapidly alleviate pain, restore limb function, and reduce tumor burden, aligning with the clinical principle of limb-sparing therapy for isolated metastatic lesions; (2) Conversion therapy strategy: This strategy is more appropriate for patients with multiple bone metastases or concomitant distant organ metastases from cholangiocarcinoma. The primary objective is to achieve tumor downstaging through systemic antineoplastic therapy, control the progression of both primary and metastatic lesions, and subsequently reassess the feasibility of local surgical intervention. In the present case, the patient’s tumor markers exhibited a marked decrease following two cycles of comprehensive antineoplastic therapy (CEA declined from 17.37 ng/mL to 11.94 ng/mL, and CA19-9 from 60.32 U/mL to 21.83 U/mL). The favorable therapeutic response suggested that the patient’s tumor was sensitive to the combination of antineoplastic therapies, including gemcitabine, albumin-bound paclitaxel, and sintilimab. Tumor progression was effectively managed, significantly reducing the risks of intraoperative tumor seeding and rapid postoperative recurrence. Given the patient’s clinical characteristics—such as isolated distal femoral metastasis, the absence of other metastatic sites, and well-controlled tumor progression following short-term systemic therapy—the multidisciplinary team (MDT) concluded that the clinical benefits of total knee arthroplasty (TKA), namely pain relief and functional reconstruction, substantially outweighed the surgical risks. This assessment confirmed the feasibility of performing TKA based on the effective control of metastatic lesions.

The mechanism underlying bone metastasis in cholangiocarcinoma may involve the hematogenous spread of tumor cells to the bone marrow microenvironment. However, due to the anatomical features of the biliary system and the tumor’s biological behavior, bone metastases predominantly occur in the blood-rich axial skeleton, with long bone metastases representing less than 5% of cases (10). Among previously documented instances of long bone metastasis from cholangiocarcinoma, there are three cases of humeral metastasis (5–7), one case of fibular metastasis (8), and a single case of femoral metastasis (9), all of which primarily presented with pathological fractures or severe pain. The patient in this case report had no discernible history of trauma and was admitted for lower limb dysfunction. Imaging revealed osteolytic lesions in the distal femur, aligning with the literature’s characterization of most long bone metastases as exhibiting osteolytic destruction (10, 11).

Patients with bone metastases from cholangiocarcinoma frequently exhibit elevated levels of CEA, CA19-9, or abnormal liver function (12). In the context of postoperative follow-up, the presence of elevated tumor markers alongside limb pain or dysfunction should raise suspicion for metastasis. Confirmation requires imaging examinations, including whole-body bone scans, computed tomography, and magnetic resonance imaging, as well as puncture biopsy pathology (13).

Although surgery remains the primary radical treatment for cholangiocarcinoma, the postoperative recurrence and metastasis rates remain high, ranging from 40% to 60%. The peak incidence of metastasis typically occurs 1 to 3 years following surgery (14). In this case, the patient developed bone metastasis 2 years postoperatively, aligning with this temporal pattern. Literature indicates that risk factors for postoperative metastasis of cholangiocarcinoma include lymph node involvement, vascular invasion, low tumor differentiation, and positive resection margins (14). The postoperative pathology for this patient revealed moderately to poorly differentiated adenocarcinoma without lymph node metastasis or vascular invasion. It is hypothesized that the metastasis may be attributed to occult hematogenous dissemination of the tumor, which also accounts for the presence of a solitary bone metastasis without involvement of other organs. P40 and P63 are established markers for squamous cell carcinoma. The pathological immunohistochemical analysis of this patient revealed partial positivity for both P40 and P63, indicating a mixed presence of adenocarcinoma and squamous cell carcinoma within the tumor tissue. This histological subtype of distal cholangiocarcinoma is rare and is associated with a poorer prognosis (15).

Bone metastases from cholangiocarcinoma are predominantly multiple, with solitary bone metastases being uncommon (10). The patient in this case presented with isolated bone metastasis, as whole-body bone scan revealed no additional metastatic sites. This characteristic supports the feasibility of limb-sparing treatment. The primary objectives for managing isolated bone metastasis are to alleviate pain, restore limb function, and extend survival. Surgical intervention can markedly enhance the patient’s quality of life (9). In cases of long bone metastasis from cholangiocarcinoma, where there is a risk of pathological fracture or limb dysfunction, the focus of surgical treatment is on limb preservation and functional reconstruction. Current surgical options include curettage with bone cement filling, intramedullary nail fixation, and prosthetic replacement (16). In this case, the distal femoral metastasis involved the articular surface, resulting in limb dysfunction and an increased risk of pathological fracture. Total knee arthroplasty was chosen, as it facilitates lesion resection, joint function reconstruction, and early weight-bearing, aligning with the principle of prioritizing limb-sparing treatment for isolated long bone metastases as recommended in the literature (9).

Kawai et al. conducted a medium- and long-term follow-up study involving 40 patients who underwent prosthesis replacement following distal femoral resection for malignant tumors. They reported 3-year and 5-year prosthesis survival rates of 85% and 67%, respectively, with 80% of patients achieving over 80% recovery of normal limb function. This finding underscores the effectiveness of prosthesis replacement in tumor limb-sparing treatments (16). In a case of cholangiocarcinoma with femoral metastasis described by MacKenzie et al. (9) the patient’s pain was significantly alleviated following distal femoral replacement, enabling him to walk independently. This further validates the clinical utility of this surgical approach in managing long bone metastasis from cholangiocarcinoma. The patient in this case experienced an uneventful recovery post-surgery, free from complications such as prosthesis loosening or infection, and was able to lead a normal life during follow-up, aligning with the significant improvement in quality of life associated with prosthesis replacement reported in the literature.

In clinical practice, postoperative cholangiocarcinoma patients should undergo routine monitoring of tumor markers and abdominal imaging. Additionally, patients experiencing limb pain and limited mobility are advised to receive timely whole-body bone scans or MRIs to facilitate the early detection of bone metastases (10). For those with isolated long bone metastases and no surgical contraindications, total joint arthroplasty represents a safe and effective treatment option that can substantially enhance the patient’s quality of life. In contrast, for patients with multiple bone metastases, the primary focus should be on palliative radiotherapy, chemotherapy, and analgesic treatment, emphasizing symptom control (11).

The prognosis for patients with bone metastases from cholangiocarcinoma is generally unfavorable, with a median survival time ranging from 3 to 13 months. This poor outcome is primarily attributed to the tumor’s high malignancy and its low responsiveness to radiotherapy and chemotherapy (11, 17). Notably,the therapeutic landscape for advanced cholangiocarcinoma is rapidly evolving, as immunotherapy and targeted therapies significantly broaden treatment options and enhance clinical outcomes for patients who previously relied on chemotherapy and radiotherapy, which often yielded poor responses (18). In the case presented, the patient exhibited no tumor recurrence during the follow-up period, which may be associated with several factors: ① the metastasis was isolated and did not involve other organs; ② timely surgical resection of the metastasis decreased the tumor burden; ③ the tumor exhibited a moderate degree of differentiation and relatively mild biological behavior. These observations indicate that for patients with isolated long bone metastases from cholangiocarcinoma, proactive surgical intervention may enhance prognosis, suggesting that local treatment should not be dismissed solely based on the overall poor prognosis of the tumor. Moreover, the positive response of this patient to sintilimab-based combination therapy reinforces the potential efficacy of immune checkpoint inhibitors (ICIs) in treating cholangiocarcinoma with bone metastasis. This finding serves as a reference for the clinical application of immunotherapy in these rare metastatic subtypes.

## Data Availability

The original contributions presented in the study are included in the article/supplementary material, further inquiries can be directed to the corresponding author.
